# The Effect of Central Injection of Ghrelin and Bombesin on Mean Plasma Thyroid Hormones Concentration

**Published:** 2011

**Authors:** Fariba Mahmoudi, Fatemeh Mohsennezhad, Homayoun Khazali, Haleh Ehtesham

**Affiliations:** a *Physiology Faculty, Payame Noor University, East Azarbaijan , Malekan Center, Iran. *; b *Physiology Faculty, Payame Noor University, Azarbijan Shargi, Malekan Center, Iran. *; c *Physiology Faculty, Shahid Beheshti University, Tehran, Iran.*; d *Biology Faculty, Payame Noor University, Iran.*

**Keywords:** Ghrelin, Bombesin, TSH, Triiodothyronine, Thyroxin

## Abstract

Ghrelin increases food intakes and body weight. Bombesin decreases food intakes and inhibits the stimulatory effect of Ghrelin on food intakes. Thyroid hormones have a crucial role in the regulation of body weight and yet the effect of bombesin on thyroid axis activity is not fully clear. Therefore, the goal of this study was to determine the effect of different doses of Ghrelin or bombesin on mean plasma thyroid-stimulating hormone (TSH), Triiodothyronine (T_3_) and Thyroxin (T_4_) concentration and also, the effect of interaction between Ghrelin and bombesin on thyroid axis activity.

Forty-nine rats in seven groups received saline or different doses of Ghrelin (4, 10 or 15 nmol) and bombesin ( 2.5, 5 or 10 nmol) and forty-two rats in six groups received simultaneous injection of Ghrelin (10 or 15 nmol) and different doses of bombesin (2.5, 5 or 10 nmol) via lateral cerebral ventricle. Blood samples were collected via decapitation 20 min after the injection and plasma was assayed for plasma TSH, T_3 _and T_4_ concentration by Radioimmunoassay (RIA).

Ghrelin significantly decreased the concentration of mean plasma thyroid hormones compared to saline. Bombesin did not significantly increase thyroid hormones concentration compared to saline but bombesin blocked the inhibitory effect of Ghrelin on thyroid axis activity. Bombesin may be the antagonist of Ghrelin action.

## Introduction

Ghrelin, a novel 28-amino acid peptide with an *n*- octanoyl modification on Ser 3, was identified in stomach as an endogenous ligand for Growth Hormone Secretagogues Receptor (GHSR-Ia) in 1999 (1, 2). Ghrelin is well recognized to have an important role in the maintenance of energy homeostasis. During fasting, Ghrelin is secreted by X/A-like cells of stomach, neurons of hypothalamus and other tissues (1-3). It increases growth hormone secretions (1), gastric empty and food intakes via GHSR-Ia (4-6). It is suggested that different peptides may interact with Ghrelin actions. Bombesin is thought to be one of these peptides (7).

Bombesin, a 14-amino acid peptide, was identified from the skin of a frog in 1970. Later, two mammalian bombesin-related peptides, Gastrin-releasing peptide (GRP) and Neuromedin B (NMB), were identified in different parts of gastrointestinal tract and brain which act via BB_1 _and BB_2_ receptors, respectively (8). Bombesin exerts its different physiological activities via BB_1_ and BB_2 _receptors (8). 

Previous studies have shown that bombesin increases the growth hormone secretion (9) and Cholecystokinin (CCK) secretion; an inhibitor hormone of food intake which blocks the stimulatory effect of Ghrelin on food intake. (10). It also decreases gastric empty (11) and suppresses food intakes (12-14). The effect of bombesin on thyroid axis activity is not completely clear. Some studies have reported an increase (15) in thyroid hormones concentration after intra-cerebral ventricle (ICV) or intra-peritoneal (IP) injection of bombesin. Furthermore, some studies reported that bombesin didn’t change thyroid hormones concentration (16, 17). It has also been reported that bombesin acts as a Ghrelin antagonist and blocks the stimulatory effects of Ghrelin on gastric motility and food intakes (7). 

Hypothalamus-pituitary-thyroid axis (HPT) plays an important role in the regulation of metabolism and energy homeostasis through thyroid hormones. It has been shown that different neural, hormonal and environmental factors interact to modulate thyroid hormones secretions. This study was designed to determine the effect of Ghrelin or bombesin on mean plasma thyroid-stimulating hormone (TSH), T_3_ and T_4_ concentration. We also designed to investigate whether bombesin might act as a Ghrelin action antagonist and might block the inhibitory effect of Ghrelin on thyroid axis activity.

## Experimental

*Animals*

Male Wistar Rats (n = 91) weighing 200-250 g (provided by the Center of Neuroscience Research of Shahid Beheshti University) were housed individually in cages under controlled temperature (22 ± 2°C) and light (12 h light/dark cycle). They had free access to food and water all the time.

*ICV cannulation and injections*

Animal surgery procedures and handling were carried out as previously described (5, 6). Animals were anesthetized by IP injection of a mixture of Ketamine and xylazine (100 mg/Kg BW Ketamine + 15 mg/Kg BW xylazine, (Alfasan Company, Holland)). For ICV injections, animals were placed in a stereotaxic frame (Stoelting, USA) and 22-gauge stainless cannulae were implanted in the right lateral cerebral ventricle. The cannulae tip was placed at anterior-posterior = - 0.8, Lateral = - 1.6 and dorsoventral = 3.2 mm according to the coordinates of Paxinos and Watson Atlas. The cannula was secured to the skull with three stainless steel screws and dental cement. The animals were kept in individual cages and habituated by handling every day to minimize the stress of surgery. After one week recovery period, 4, 10 or 15 nmol of Ghrelin or 2.5, 5 or 10 nmol of bombesin (Ghrelin and bombesin provided by Ana Spec Company (Ana Spec, USA)) were dissolved in 5 μL of 0.9% saline. The peptides were injected by a 27-gauge stainless steel injector which connected to 10 μL Hamilton micro syringe (model 9435, Australia) by PE-20 tubing. At the end of the experimentation, the animals were decapitated and the blood samples were collected 20 min after the injections. The dosage of the peptides and the time of blood sampling were chosen based on the previous experiments (5, 6, 15). Heparin was used in samples to prevent clotting. Blood samples immediately centrifuged for 10 min at 3500 rpm and the plasma stored at–20°C until TSH, T_3_ and T_4_ concentrations were assayed. The animals’ brains were removed and kept in formalin (10%) for two weeks. The proper ICV cannulae placement was confirmed histologically. Only those animals with properly-positioned cannulae were included in data analysis.

Initially, the effect of different doses of either Ghrelin or bombesin on thyroid axis activity was investigated. Forty-nine rats in 7 groups (in each group, n = 7) received saline, Ghrelin (4, 10 or 15 nmol) or bombesin (2.5, 5 or 10 nmol) in a volume of 5 μL over one min during the early light phase (0800 h - 0900 h). Blood samples were collected by decapitation 20 min after the injections. In each group, the mean plasma TSH, T_3_ and T_4_ concentration were measured. The effect of simultaneous administration of Ghrelin and bombesin was examined afterwards. Forty- two rats in six groups (in each group, n = 7) received simultaneous administration of Ghrelin (10 or 15 nmol) and bombesin (2.5, 5 or 10 nmol) in a volume of 5 μL during the early light phase (0800 h - 0900 h). Blood samples were collected by decapitation 20 min after injections. In each group, the mean plasma TSH, T_3_ and T_4 _concentration were measured.

*Hormone assays and statistical analysis*

Plasma TSH, T3 and T_4 _were measured by using TSH, T_3_ and T_4_ kits (Sahand Iran Company, Iran) and the method of a homologous double antibody radio-immunoassay (RIA). The results are presented as mean ± SEM The data were analyzed by unpaired t-test, ANOVA test followed by post hoc Least Significant Difference and SPSS software. In all cases, p < 0.05 was considered to be statistically significant.

## Results and Discussion

**Figure 1:**
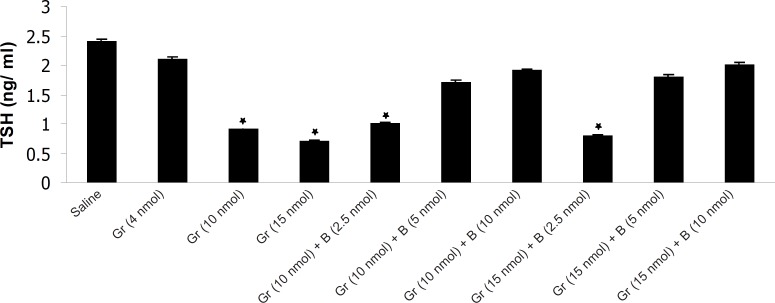
The effect of different doses of Ghrelin (Gr) and the effect of simultaneous administration of Ghrelin (Gr) and different doses of bombesin (B) on mean plasma TSH compared to saline (p < 0.05). In comparison with saline, Ghrelin (10 or 15 nmol) significantly decreased the mean plasma TSH concentration and Bombesin (5 or 10 nmol) significantly blocked the inhibitory effect of Ghrelin on mean plasma TSH (p < 0.05).

**Figure 2:**
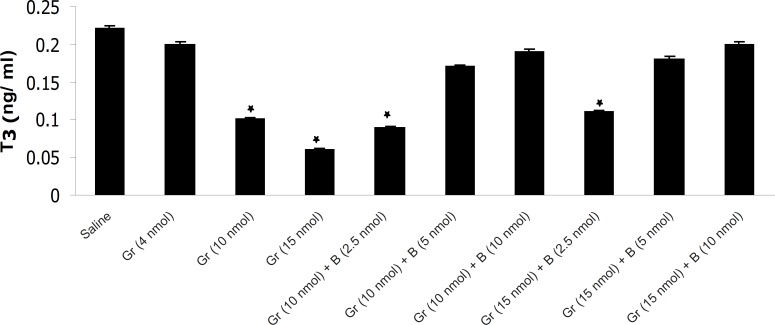
The effect of different doses of Ghrelin (Gr) and the effect of simultaneous administration of Ghrelin (Gr) and different doses of bombesin (B) on mean plasma T_3_ compared to saline (p < 0.05). In comparison with saline, Ghrelin (10 or 15 nmol) significantly decreased the mean plasma T_3 _concentration and Bombesin (5 or 10 nmol) significantly blocked the inhibitory effect of Ghrelin on mean plasma T_3_ (p < 0.05).

**Figure 3:**
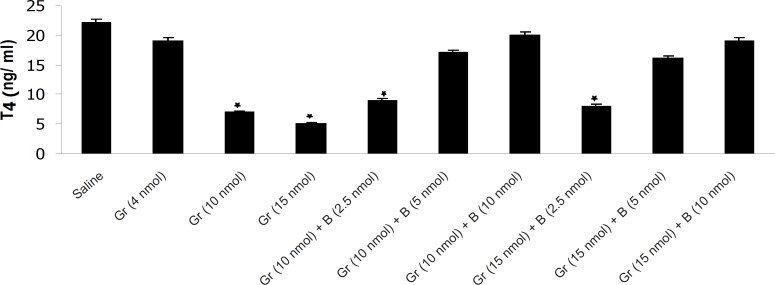
The effect of different doses of Ghrelin (Gr) and the effect of simultaneous administration of Ghrelin (Gr) and different doses of bombesin (B) on mean plasma T_4_ compared to saline (p < 0.05). In comparison with saline, Ghrelin (10 or 15 nmol) significantly decreased the mean plasma T_4_ concentration and Bombesin (5 or 10 nmol) significantly blocked the inhibitory effect of Ghrelin on mean plasma T_4_ (p < 0.05

The results have shown that Ghrelin significantly decreased mean plasma TSH, T_3_ and T_4_ concentration compared to saline. Ghrelin (4 nmol) did not significantly alter mean plasma thyroid hormones concentration compared to saline but Ghrelin (10 or 15 nmol) significantly decreased TSH, T_3_ and T_4_ compared to saline (Figures 1, 2 and 3). Also as it is shown in Table 1, different doses of bombesin increased the mean plasma TSH, T_3_ and T_4_ concentration compared to saline but this increase was not statistically significant (Table 1).

**Table 1:** The effect of different doses of just Ghrelin (Gr), just bombesin (B) and simultaneous injection of Ghrelin and different doses of bombesin on mean plasma TSH, T_3_ and T_4 _concentration compared to saline

		**TSH**	**T**_3_	**T**_4_
**Gr (4 nmol)**	12%		10%	14%
**Gr (10 nmol)**	62%		55%	68%
**Gr (15 nmol)**	71%		73%	77%
**B (2.5 nmol)**	8%		9%	14%
**B (5 nmol)**	21%		22%	23%
**B (10 nmol)**	33%		32%	36%
**Gr (10 nmol) + B (2.5 nmol) **		58%	59%	
**Gr (10 nmol) + B (5 nmol)**	59%		23%	23%
**Gr (10 nmol) + B (10 nmol)**	21%		14%	10%
**Gr (15 nmol) + B (2.5 nmol)**	67%		50%	64%
**Gr (15 nmol) + B (5 nmol)**	25%		18%	27%
**Gr (15 nmol) + B (10 nmol)**	17%		9%	14%

The results also showed that in comparison with saline, Ghrelin (2.5 nmol) does not abolish the inhibitory effect of bombesin on mean plasma TSH, T_3_ and T_4_. In contrast, bombesin (5 or 10 nmol) significantly blocked the inhibitory effect of Ghrelin on mean plasma TSH, T_3_ and T_4_ compared to saline (Figures 1, 2 and 3).

The results of this study showed that ICV injection of bombesin didn’t significantly alter the thyroid hormones concentration which is consistent with the previous studies (16, 17). Moreover, the results showed that Ghrelin significantly decreased the mean plasma TSH, T_3_ and T_4_ concentrations.

It seems that the significant fall in TSH, T_3_ and T_4_ concentrations after the infusion of Ghrelin was most likely due to the effect of Ghrelin on different peptides (like Agouti-related peptide (AgRP) or Neuropeptide Y (NPY)) in the arcuate nucleus (ARC) of hypothalamus.

Previous studies have demonstrated that Ghrelin increases the synthesis of AgRP/ NPY in ARC neurons (4-6). It is also established that AgRP/ NPY- immunoreactive axons densely innervates the thyrotropin-releasing hormone (TRH) neurons in the paraventricular nucleus (PVN) of hypothalamus and the exogenous ICV or PVN infusion of AgRP or NPY markedly inhibits the H-P-T axis activity (18-21). So, Ghrelin may have an inhibitory effect on thyroid axis activity via increasing AgRP or NPY. 

Furthermore, it has been found that AgRP acts as an endogenous antagonist or inverse agonist at melanocortin receptors including MC_3_ and MC_4_ receptors on TRH neurons. The studies have shown that *α*-melanocyte-stimulating hormone (*α*MSH) neurons of ARC densely innervate the TRH neurons of PVN and there is a significant increase in TSH and thyroid hormones level after ICV or PVN injection of *α*MSH (18, 22, 23). Therefore, we could expect that inhibitory activity of Ghrelin on hypothalamic-pituitary-thyroid (HPT) axis, at least partially, may due to an increase in the AgRP level and its antagonist action on *α*MSH receptors.

It has been suggested that central Ghrelin blocked the GABA release from AgRP or NPY neurons of hypothalamus. The inhibition of GABA release is involved in the activation of CRF neurons and the increasing in corticotrophin releasing hormone (CRH) from hypothalamus. (24). As the CRH and cortisol exert an inhibitory effect on plasma TSH, T_3_ and T_4 _concentrations (25, 26), the inhibitory effect of Ghrelin on thyroid axis may be partially due to the stimulatory effect of it on hypothalamus-pituitary-adrenal (HPA) axis. 

In the present study, the effect of interaction between Ghrelin and bombesin on thyroid axis activity was investigated for the first time. The results demonstrated that bombesin significantly blocked the inhibitory effect of Ghrelin on mean plasma TSH, T_3_ and T_4_ concentration.

However, previous studies have been showed that bombesin blocks the stimulatory effect of Ghrelin on gastric motility and food intakes (7). This study has showed that bombesin blocks the inhibitory effect of Ghrelin on thyroid axis activity. Further studies are needed to determine the possibility of the effect of bombesin on Ghrelin actions. 

## Conclusion

Ghrelin significantly decreased the mean plasma TSH, T_3_ and T_4_ concentrations and bombesin blocks the inhibitory effect of Ghrelin on thyroid axis activity. The results suggested that bombesin may be the antagonist of Ghrelin action.
